# Nuclear Translocation of Cardiac G Protein-Coupled Receptor Kinase 5 Downstream of Select Gq-Activating Hypertrophic Ligands Is a Calmodulin-Dependent Process

**DOI:** 10.1371/journal.pone.0057324

**Published:** 2013-03-05

**Authors:** Jessica I. Gold, Jeffrey S. Martini, Jonathan Hullmann, Erhe Gao, J. Kurt Chuprun, Linda Lee, Douglas G. Tilley, Joseph E. Rabinowitz, Julie Bossuyt, Donald M. Bers, Walter J. Koch

**Affiliations:** 1 Center for Translational Medicine, Thomas Jefferson University, Philadelphia, Pennsylvania, United States of America; 2 Center for Translational Medicine, Temple University School of Medicine, Philadelphia, Pennsylvania, United States of America; 3 Department of Pharmacology, Temple University School of Medicine, Philadelphia, Pennsylvania, United States of America; 4 Department of Pharmacology, University of California Davis, Davis, California, United States of America; University of Illinois at Chicago, United States of America

## Abstract

G protein-Coupled Receptors (GPCRs) kinases (GRKs) play a crucial role in regulating cardiac hypertrophy. Recent data from our lab has shown that, following ventricular pressure overload, GRK5, a primary cardiac GRK, facilitates maladaptive myocyte growth via novel nuclear localization. In the nucleus, GRK5’s newly discovered kinase activity on histone deacetylase 5 induces hypertrophic gene transcription. The mechanisms governing the nuclear targeting of GRK5 are unknown. We report here that GRK5 nuclear accumulation is dependent on Ca^2+^/calmodulin (CaM) binding to a specific site within the amino terminus of GRK5 and this interaction occurs after selective activation of hypertrophic Gq-coupled receptors. Stimulation of myocytes with phenylephrine or angiotensinII causes GRK5 to leave the sarcolemmal membrane and accumulate in the nucleus, while the endothelin-1 does not cause nuclear GRK5 localization. A mutation within the amino-terminus of GRK5 negating CaM binding attenuates GRK5 movement from the sarcolemma to the nucleus and, importantly, overexpression of this mutant does not facilitate cardiac hypertrophy and related gene transcription *in vitro* and *in vivo*. Our data reveal that CaM binding to GRK5 is a physiologically relevant event that is absolutely required for nuclear GRK5 localization downstream of hypertrophic stimuli, thus facilitating GRK5-dependent regulation of maladaptive hypertrophy.

## Introduction

Canonically, G protein-coupled receptor (GPCR) kinases (GRKs) desensitize GPCRs via agonist-dependent phosphorylation. Seven members of the GRK family have been identified to date with GRK2 and GRK5 being the most abundant in the heart [1], [2]. These kinases have been shown to play important roles in physiological cardiac signaling, particularly via regulation of β-adrenergic receptor (βAR)-mediated contractility [2]–[5]. GRK2 and GRK5 appear to be critical in cardiac pathophysiology [2], [3], as upregulation of both GRK2 and GRK5 has been shown in a spectrum of cardiac pathology including failing human myocardium [1], [6]–[11]. Despite similar functions in GPCR desensitization, increased expression of GRK2 and GRK5 play divergent roles in compromised myocardium during the pathogenesis of heart failure (HF). Utilization of genetically engineered mouse models has been key to understanding how GRK2 and GRK5 elevation lead to distinct cardiac phenotypes. For example, transgenic mice with cardiac-specific overexpression of GRK5 demonstrate intolerance to ventricular pressure-overload, as evidenced by augmented cardiac hypertrophy and accelerated HF following aortic banding [12]. This accelerated pathological phenotype differs greatly from mice overexpressing GRK2, which respond to pressure-overload similarly to wild-type mice [12]. This phenotypic disparity is rooted in differences between the structure and subcellular localization of GRK2 and GRK5, predominantly the ability of GRK5 to enter the nucleus [12]–[15].

Among GRK family members, GRK5’s ability to enter the nucleus is unique. First shown in cardiomyocytes of spontaneously hypertensive HF (SHHF) rats, the ability of GRK5 to translocate to the nucleus was further reinforced by uncovering a nuclear localization sequence (NLS) within its catalytic domain [13]–[15]. We recently identified the first nuclear target of GRK5 activity – the class II histone deacetylase (HDAC), HDAC5, which occurs after GRK5 nuclear accumulation following in vivo and in vitro hypertrophic stimuli mediated via Gq-coupled signaling activation [12]. Like other known class II HDAC kinases [16]–[19], enhanced nuclear GRK5 activity increases transcription of genes associated with cardiac hypertrophy, through derepression of critical transcription factors [20]. Most important among these transcription factors is myocyte enhancer factor 2 (MEF2), the upstream regulator of several hypertrophic genes [16], [21], [22].

This expanding range of substrates is coupled to greater complexity of the kinase’s regulation, particularly in light of GRK specificity for distinct receptors. For example, each GRK can directly interact with Ca^2+^ binding proteins *in vitro* [23]. These interactions tend to decrease kinase activity at the receptor [24]. Ca^2+^/Calmodulin (CaM) is able to bind all GRK family members, but with varying affinities [25]. CaM preferentially binds GRK5 (IC_50_∼50 nM) at a CaM binding domain in either terminal domain [25]. Once CaM-bound, particularly at the amino (N)-terminal site, GRK5 demonstrates decreased kinase activity at the receptor and activity at cytosolic substrates including synuclein and tubulin [26]. Alternatively, phosphorylation by PKC at a carboxy (C)-terminal site inhibits GRK5’s activity against all substrates, membrane-bound and cytosolic [27]. Despite growing interest in GRK regulation, corresponding *in vivo* studies demonstrating physiological relevance have been scarce.

In this study, our goal was to uncover the molecular mechanisms responsible for GRK5 nuclear localization during hypertrophic Gq activation and signaling in myocytes. Understanding the mechanism behind nuclear translocation of GRK5 could present a novel therapeutic target for prevention of maladaptive cardiac remodeling. This is especially important because although we have shown nuclear GRK5 to be pathologic, GRK5 action at the plasma membrane has shown to be cardioprotective under certain circumstances [28]. Here, we show that select hypertrophic agonists of Gq-coupled receptors cause GRK5 nuclear translocation from a plasma membrane pool in myocytes. These specific ligands target CaM binding to N-terminal residues within GRK5 that we demonstrate to be an absolute requirement for nuclear translocation and GRK5-mediated pathological cardiac signaling. Targeted inhibition of CaM binding to GRK5 leads to less nuclear accumulation, activity and hypertrophic signaling and, interestingly, greater GRK5 retention at the membrane, even after GPCR activation. Of note, we find an *in vivo* pathophysiological link between a direct CaM-GRK5 interaction and maladaptive cardiac hypertrophy. This increased understanding of the pathological mechanisms of nuclear GRK5 activity provides a potential therapeutic target to limit cardiac maladaptation while potentially preserving beneficial GPCR-desensitizing properties.

## Materials and Methods

### Reagents

PE, AngII, ET-1, Iso, CDZ, W-7, Bis1, Go6976, KN-93 were all purchased from Sigma Aldrich. 2-APB and Adenophostin were acquired from Calbiochem. Antibodies used against GRK5 were either from Millipore (05–466) or Santa Cruz (sc-565). Anti-fibrillarin was purchased from Cell Signaling (C13C3). Anti-GAPDH was from Chemicon (MAB374). β-tubulin was acquired from Abcam (ab40862).

### Cell Culture and Adenoviral Infection

All animal procedures and experiments were performed in strict accordance with the guidelines of the Institutional Animal Care and Use Committee (IACUC) of Thomas Jefferson University under IACUC-approved protocol 731W. All surgery was performed under isoflurane anesthesia, and all efforts were made in minimize suffering. Our euthanasia method was inhalation of 100% carbon dioxide followed by cervical dislocation. Ventricular cardiomyocytes were isolated from 1- to 2-day old neonatal rat hearts (NRVM) as previously described [29]. NRVM were cultured in DMEM supplemented with penicillin/streptomycin (100 units/ml) and 5% FBS at 37°C in a 5% humidified atmosphere for 2–3 days. At 24 hrs post-isolation, NRVM were infected with recombinant, replication-deficient adenoviruses expressing the following genes with their respective MOIs: GRK5 (50 MOI), Gq-CAM (5 MOI), GRK5W30A (15 MOI). Equal particles of an adenovirus expressing LacZ were used to control for non-specific adenoviral effects. NRVM were serum-starved for 24 hours prior to harvest in DMEM supplemented with penicillin/streptomycin and.5% FBS at 37°C in a 5% humidified atmosphere. AdRbM were isolated as described elsewhere [18]. Myocytes were seeded on lamin-coated chamber slides and cultured in supplemented PC-1 with penicillin/streptomycin. Four hours after seeding, myocytes were infected with adenoviruses expressing either GRK5-GFP (100 MOI) or GRK5W30A-GFP (200 MOI) and cultured for 24 hours prior to experimentation.

### Western Blotting

Western blots for GRK5 (05–466, Millipore), fibrillarin (C13C3, Cell Signaling), β-tubulin (ab40862, Abcam) and glyceraldehyde-3-phosphate dehydrogenase (GAPDH) (MAB374; Chemicon) were performed as described previously using protein extracts from cell lysates [12]. Visualization of Western blot signals was performed using secondary antibodies coupled to Alexa Fluor 680 or 800 (Molecular Probes) on a LI-COR infrared imager (Odyssey). Pictures were processed by Odyssey version 1.2 infrared imaging software. All densitometry scans were carried out in the linear range of detection.

### Immunofluorescence

Myocytes were fixed on glass coverslips using 4% paraformaldehyde as previously described [12]. Membranes were permeabilized using a.1% Triton X buffer. Cells were washed and blocked using.5% BSA. Primary antibodies for GRK5 (sc-565, Santa Cruz) were added at 1∶1,000. Secondary antibodies were conjugated to AlexaFluor 488 or 568 (Invitrogen).

### TIRF (Total Internal Refraction Fluorescence Microscopy)

An argon laser light (488 nm) was directed through the objective with a multiple band dichroic mirror. TIRF emission was selected with a filter of 515/30 nm for GFP [30]. Filter transitions and shutter events were automated with MetaMorph acquisition software. Myocytes were imaged every 10 seconds for 12 minutes. Ligand was added 120 seconds after imaging was initiated. At least 30 cells from 4 adult rabbit isolations were imaged for each group.

### Cellular Fractionation

Cellular fractionation in the NRVM was performed as previously described [31]. Cellular fractionation from cardiac tissue was modified from the referenced procedure. Isolated tissue was first homogenized using a Dounce homogenizer in a buffer containing: 4 mM Hepes, 320 mM sucrose, 10 mM KCL, 5 mM EDTA, 2 mg NaF, 8 mg MgCl_2_,.1% Triton x-10, 1.094 g DTT, and protease inhibitors. The homogenate was filtered through a 70 µm cell filter. Total cell lysate was taken at this point. Then the lysate was subjected the same protocol as that for the NRVM.

### Mini-osmotic Pumps

Chronic infusion of hypertrophic ligands of Gq-coupled receptors was achieved using Alzet 3-day mini-osmotic pumps (model 1003D, DURECT Corporation). Pumps were filled following the manufacturer’s specifications with sterile PBS, PE (30 µM/kg/day), AngII (200 nM/kg/min) and Iso (60 mg/kg/day). Briefly, Mice were anesthetized with isoflurane (2.5% vol/vol) and pumps were implanted subcutaneously through a sub-scapular incision, which was then closed using 4.0 silk suture (Ethicon). The contents of the pumps were delivered at a rate of 1.0 µl/hour for 3 days. Mice were monitored daily and euthanized on day 3.

### Echocardiography

Echocardiography was performed as previously described [29]. To measure global cardiac function, echocardiography was performed at 8 weeks of age prior to mini-osmotic pump implantation and 72 hours following pump implantation by use of the VisualSonics VeVo 770 imaging system with a 707 scan head in anesthetized animals (1.5% isoflurane, vol/vol). The internal diameter of the left ventricle was measure in the short-axis view from M-mode recordings in end diastole and end systole.

### Confocal Imaging

GRK5-GFP and GRK5W30A-GFP signals were measured by confocal microscopy using argon laser excitation at 488 nm and emitted fluorescence at LP 500. Data were analyzed using Image J software with the intensity of the regions of interest (ROI) normalized to area. ROI measurements were also corrected for background signal [18]. At least 30 cells were imaged per group from 3 adult rabbit isolations.

### Luciferase Assay

Cells were harvested 48 hrs after infection in passive lysis buffer (Promega). Luciferase activity was measured according to manufacturer’s protocol (Promega) using a Victor plate reader. Luciferase units were normalized to total protein [12].

### Measurement of IP_3_

IP_3_ generation can be measured by the stable accumulation of IP_1_ in cells in the presence of LiCl following agonist binding to Gq-coupled receptors [32]. IP_1_ measurements were performed by ELISA (Cisbio), according to the manufacturer’s protocol, and optical density at 450 nm was read using a Victor plate reader.

### Myocardial Gene Delivery

Adenoviruses expressing either GRK5W30A or GRK5 CTPB were delivered as previously described with minor changes [33]. Briefly, 8 week-old global GRK5KO mice were anesthetized with 2% isoflurane inhalation and not ventilated. A skin cut (1.2 cm) was made over the left chest and a purse suture was made. After dissection of pectoral muscles and exposure of the ribs, the heart was smoothly and gently “popped out” through a small hole made at the 4th intercostal space. Each adenovirus was diluted to 2.5×10^11^ particles and 25 µl was then injected directly into LV free wall with a Hamilton syringe (Hamilton Co. Reno, Nevada) with the needle size of 30.5). Three points injections are performed: 1) starting from apex and moving toward to the base in LV anterior wall; 2) at the upper part of LV anterior wall; and 3) starting at the apex and moving toward to base in LV posterior wall. After the gene delivery, heart was immediately placed back into the intrathoracic space followed by manual evacuation of pneumothoraces and closure of muscle and the skin suture.

### Statistics

All the values in the text and figures are presented as mean ± SEM from at least three independent experiments from given *n* sizes. Statistical significance of multiple treatments was determined by one-way ANOVA followed by the Bonferroni’s post hoc test when appropriate. Statistical significance between two groups was determined using the two-tailed Student’s *t* test. *P* values of <0.05 were considered significant.

## Results

### Determining a Physiological Stimulus for Nuclear GRK5 Translocation

**Figure 1:**
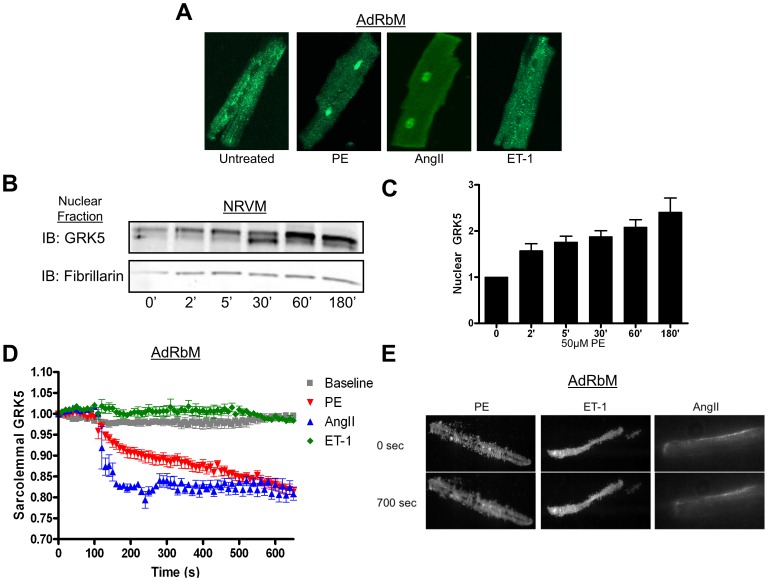
PE and AngII induce translocation of GRK5 from the membrane to the nucleus. (**A**) Representative immunofluorescence staining of endogenous GRK5 in AdRbM shows increased nuclear GRK5 following PE (50 µM) and AngII (10 µM) treatment, but not ET-1 (100 nM). (**B**) NRVM were infected with Ad-GRK5 (50 MOI). After 48 hr, cells were treated with 50 µM PE for 5 different time points, harvested by subcellular fractionation. Nuclear were fractions immunoblotted for GRK5 and fibrillarin. (**C**) The amount of GRK5 in the nucleus was calculated by denistometry, normalized to fibrillarin, and reported as Fold Change over baseline. *p<0.05, one-way ANOVA with a Bonferroni correction, n = 4. (**D**) Rabbit myocytes were infected with an adenovirus expressing GRK5-GFP and cultured overnight. Using TIRFM cells were imaged at 10 sec intervals for 700 sec. Baseline myocytes were untreated while stimulated myocytes were treated with either PE (50 µM), AngII (10 µM), or Et-1 (100 nM) at 120 sec. Fluorescence was normalized and reported as fold change versus baseline. n = 4. (**E**) Representative TIRF images for each agonist at the beginning and end of imaging.

The nuclear localization of GRK5 in cardiac myocytes has been shown previously under generalized stress, such as in SHHF rats [14], [15], post-transverse aortic constriction (TAC) in mice, or *in vitro* by infecting myocytes with an adenovirus expressing a constitutively active Gαq (Gq-CAM) subunit [12]. Regardless of the model, these findings all show that GRK5 localizes to the nucleus downstream of Gq, the nodal signaling trigger for pathological hypertrophy [34]–[36]. Due to qualitative similarities, it is possible to use these varied models in a complementary fashion. To further advance our understanding of hypertrophic agonist-induced nuclear GRK5 localization, we investigated select Gq-coupled receptor ligands known to induce cardiac hypertrophy. Specifically, we tested phenylephrine (PE), endothelin-1 (ET-1), and angiotensinII (AngII). Immunostaining was used to assess the subcellular localization of GRK5 in adult rabbit ventricular myocytes (AdRbM) following stimulation with PE (50 µM), ET-1 (100 nM) or AngII (10 µM). Data in Fig. 1A show that PE and AngII can induce GRK5 translocation to the nucleus of adult rabbit ventricular myocytes (AdRbM), while ET-1 does not lead to nuclear accumulation of this kinase.

The ability of PE and AngII to cause nuclear translocation of GRK5 was further studied in neonatal rat ventricular myocytes (NRVM). Overexpression of GRK5 in these cells results in significant basal levels of GRK5 in the nucleus (On-line Fig. S1A). In comparison, endogenous *in vivo* cardiac GRK5 is normally present at low levels in the nucleus with the majority of the kinase non-nuclear (Fig. 1A and on-line Fig. S1B). In NRVM, additional nuclear accumulation of GRK5 was measured after PE treatment. At time-points of 30 min and longer, PE stimulation led to significantly greater nuclear GRK5 levels with an increase to 241±30% of baseline by 180 min (Fig. 1B, C). AngII treatment of NRVM resulted in a similar increase (On-line Fig. S2A). In contrast, treatment of NRVM with ET-1 over the same period caused no change in nuclear GRK5 (On-line Fig. S2B). NRVM were also treated with isoproterenol (Iso) (10 µM), a drug that can cause myocyte growth through Gs-coupled βARs. However, this ligand did not increase nuclear GRK5 (On-line Fig. S2C). The lack of Iso-induced nuclear GRK5 suggests that GRK5’s nuclear translocation lies solely downstream of Gq-coupled GPCRs, specifically the α-adrenergic receptor (α_1_AR) and the AngII receptor (AT_1_R).

Classically, GRK5 has been shown to be strongly associated with the plasma membrane [2], [37], which is consistent with our findings in AdRbM (Fig. 1A and On-line Fig. S1B, C). One question we wanted to address was whether the accumulated GRK5 in the nucleus after PE and AngII treatment was related to the pool of GRK5 at the plasma membrane. To address specific movement of membrane-bound GRK5, we used total internal reflection fluorescence (TIRF) microscopy. AdRbM were infected with an adenovirus expressing a GFP-tagged GRK5 and imaged every 10 sec for 700 sec. At 120 sec, treated cells received the above agonists (PE, ET-1, or AngII) at given concentrations. In cardiomyocytes treated with PE or AngII, we found a swift and sustained decrease in fluorescence, signifying movement of GRK5 away from the membrane (Fig. 1D, E). Interestingly, myocytes treated with ET-1 showed no change in fluorescence over basal measurements, indicating that this Gq-coupled receptor agonist does not cause translocation of GRK5 from the plasma membrane. The specificity of TIRF data from these hypertrophic agonists’ shows a correspondence between loss of GRK5 at the plasma membrane and nuclear accumulation of the kinase suggesting that nuclear GRK5 originates from the membrane pool.

### Defining the Role of PE and AngII on Nuclear GRK5 *in vivo*

**Figure 2:**
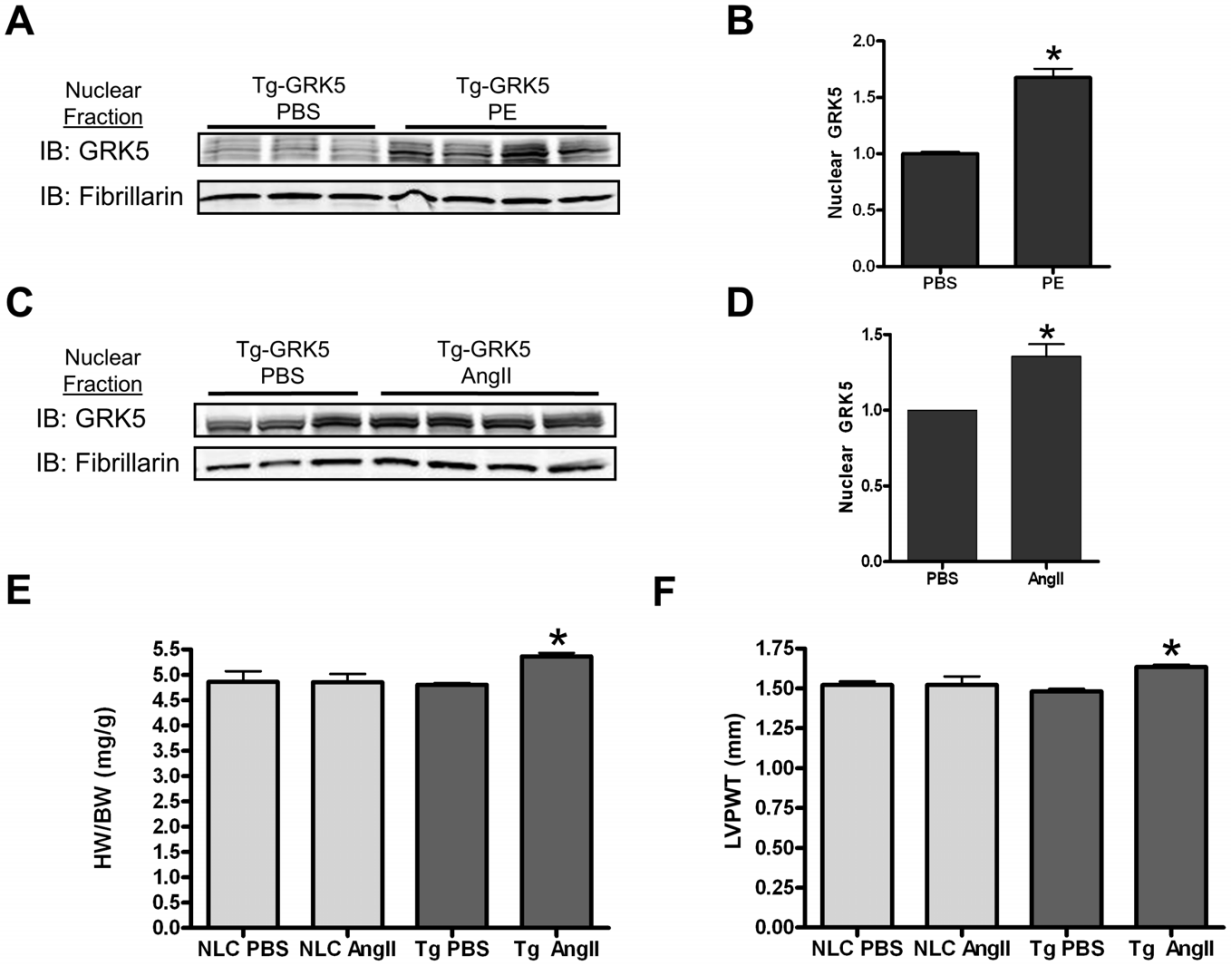
Mice with cardiac-overexpression of GRK5 (Tg-GRK5) show increased nuclear accumulation of GRK5 following 3 days of continuous infusion of a subpressor dose of PE or AngII. (**A**) Osmotic minipumps containing either a subpressor dose of PE (30 µM/kg/day) or phospho-buffered saline (PBS) were implanted subcutaneously in Tg-GRK5 mice. After 72 hr, hearts were isolated and subjected to subcellular fractionation and immunoblotted for GRK5 and fibrillarin. (**B**) The amount of GRK5 in the nucleus was calculated by denistometry, normalized to fibrillarin, and reported as the fold change increase with PE. *p<0.001 v. PBS treated, student’s t-test, n = 8. (**C**) Osmotic minipumps containing either a subpressor dose of AngII (200 nM/kg/min) or PBS were implanted subcutaneously in Tg-GRK5 mice. After 72 hr, hearts were isolated and subjected to subcellular fractionation and immunoblotted for GRK5 and fibrillarin. (**D**) The amount of GRK5 in the nucleus was calculated by denistometry, normalized to fibrillarin, and reported as the fold change increase due to AngII. *p<0.01 v. PBS treated, student’s t test, n = 9. (**E**) HW/BW ratio following 3 days of continuous PBS or AngII infusion in NLC and Tg-GRK5. *p<0.01 v. Tg PBS and NLC AngII, one-way ANOVA with a Bonferroni correction, n = 5–9 (F) Systolic LV Posterior Wall thickness (LVPWT) measured in mm by echocardiogram following 3 days of continuous PBS or AngII infusion in NLC or Tg-GRK5 mice. *p<0.01 v. Tg PBS and NLC AngII, one-way ANOVA with a Bonferroni correction, n = 5–9.

The above results examined the role of Gq-coupled agonists in adult and neonatal myocytes. We were also interested in determining the role of PE and AngII *in vivo*. Here, we utilized our transgenic mice with cardiac-specific overexpression of GRK5 (Tg-GRK5) [12], [38]. Male Tg-GRK5 mice or non-transgenic littermate control (NLC) mice were subjected to three days of chronic infusion of a subpressor dose of PE (30µ M/kg/day) or AngII (200n M/kg/day) via implanted osmotic minipumps. Control Mice were infused with phospho-buffered saline (PBS). Cardiac function and dimensions were measured by echocardiogram prior to pump implantation and at the end of the 72 hr period. Importantly, after fractionation of homogenized hearts, we found that 3 days of PE or AngII treatment led to significantly elevated GRK5 levels in the nuclear fraction (Fig. 2A–D). As a further control for Gq-specific GRK5 nuclear translocation, 3 days of Iso (60 mg/kg/day) treatment in Tg-GRK5 mice did not lead to increased GRK5 levels in the nucleus of myocytes (On-line Fig. S3). Surprisingly, we found that after only 3 days of treatment with AngII, Tg-GRK5 mice had slight, but significant cardiac hypertrophy. This was evidenced by increased heart weight-to-body weight (HW/BW) ratios (4.81±0.057 mg/g PBS-infused vs. 5.363±0.138 mg/g AngII-infused, p<0.01) (Fig. 2E), and increased left ventricular (LV) posterior wall thickness during systole (1.48±0.038 mm vs. 1.635±0.035 mm, PBS- and AngII-infused, respectively, p<0.001) (Fig. 2F). Notably, this dose and treatment schedule of AngII in NLC mice did not lead to increased cardiac size (Fig. 2E–F), indicating that AngII-driven nuclear GRK5 seen in Tg-GRK5 mice can induce and potentiate cardiac hypertrophy.

### Mechanistic Role of CaM in Gq-Mediated GRK5 Nuclear Translocation

**Figure 3:**
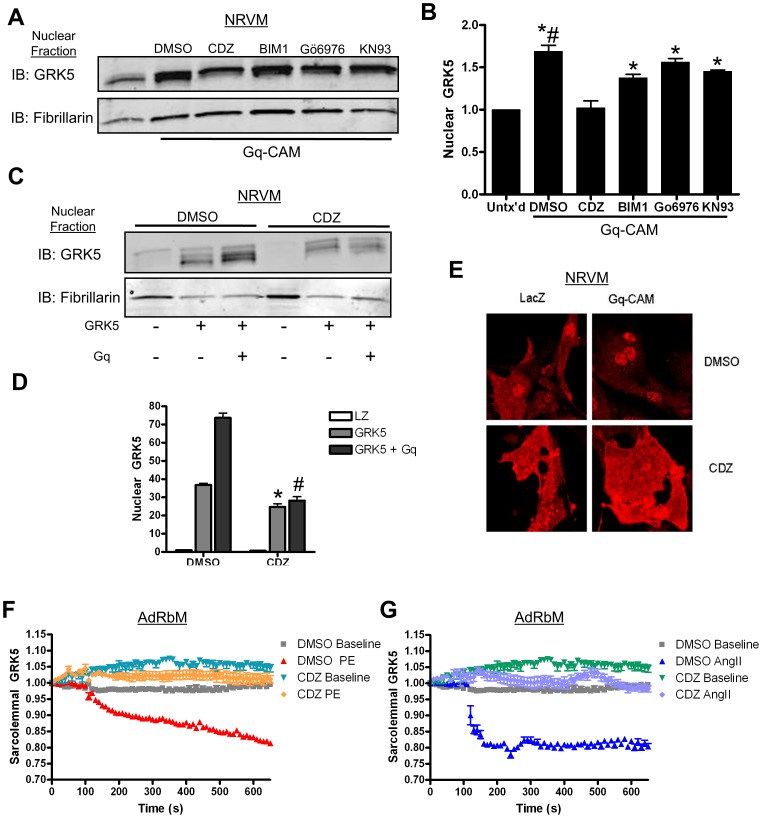
GRK5 nuclear accumulation is diminished after treatment with a CaM inhibitor. (**A**) NRVM were infected with Ad-GRK5 and either Ad-LacZ or Ad-Gq-CAM. 48 hr after infection, cells were treated with DMSO or inhibitor: BIM1 (10 µM), Gö6976 (10 µM), CDZ (10 µM) and KN93 (10 µM) for 1 hr. The cells were harvested using subcellular fractionation and immunoblotted for GRK5. (**B**) Immunoblots were quantitated by densitometry, normalized to fibrillarin, and reported as fold change over baseline. * p<0.05 v. untreated baseline, # p<0.05 v. CDZ, one-way ANOVA with a Bonferroni correction, n = 4. (**C**) NRVM were infected with Ad-LacZ, Ad-GRK5 and Ad-Gq-CAM. 48 hr after infection, cells were treated with DMSO or CDZ (10 µM) for 1 hr. The cells were harvested using subcellular fractionation, and immunoblotted for GRK5. (**D**) Densitometric analysis for (**C**) with GRK5 normalized to fibrillarin and calculated as fold change over baseline. *p<0.01 v. DMSO GRK5, #p<0.01 v. DMSO GRK5+ Gq, one-way ANOVA with a Bonferroni correction, n = 4. (**E**) NRVM were infected with either Ad-LacZ or Ad-Gq-CAM. 48 hr after infection, cells were treated with DMSO or CDZ (10 µM). Immunofluorescence was detected using a polyclonal GRK5 antibody. (**F**) TIRF analysis of AdRbM infected with an adenovirus expressing GRK5-GFP and cultured overnight. Cells were imaged at 10 sec intervals for 700 sec. Cells were pre-treated with CDZ or DMSO for 30 min at 37°C prior to imaging. Baseline myocytes were untreated while stimulated myocytes were treated with PE (50 µM) at 120 sec. Fluorescence was normalized and reported to fold change versus baseline. n = 4. (**G**) Same experimental design as (**F**) except cells were stimulated with AngII (10 µM) at 120 s. n = 4.

Having established specific ligands upstream of Gq leading to physiologically relevant movement of GRK5 to the nucleus of myocytes, we turned our attention to potential downstream mechanisms. We initially identified a handful of downstream effectors of Gq signaling that have been shown to interact with GRK5: PKC, CaM, PKD and CaMKII [25]–[27]. Inhibitors targeting these effectors were utilized to elucidate any potential role in GRK5’s nuclear translocation following Gq activation. We co-infected NRVM with a GRK5-containing adenovirus (Ad-GRK5) and the Gq-CAM adenovirus (Ad-Gq-CAM). After 48 hrs of infection, we treated cells for 1 hr with either DMSO, as the control vehicle, or various inhibitors (targets listed in parenthesis): BIM1 (PKC), CDZ (CaM), Gö6976 (PKD), or KN-93 (CaMKII). Nuclear levels of GRK5 were then determined (Fig. 3A). As expected, Gq-CAM increased nuclear GRK5 levels significantly over basal conditions (68.9±14.3%). As shown in Fig 3A–B cells infected with Gq-CAM and treated with BIM1, Gö6979 and KN-93 also showed significantly increased nuclear GRK5 compared to baseline. However, myocytes infected with Gq-CAM and treated with CDZ showed no significant rise in nuclear GRK5 levels over baseline (2.1±16.7%, p = NS vs. untreated). CDZ inhibition of CaM also led to a significant decrease in nuclear GRK5 compared to cells expressing Gq-CAM and treated with DMSO (Fig 3A, B). Importantly, this experiment was repeated using PE. After 1 hr of α_1_AR stimulation, myocytes showed an increased level of nuclear GRK5 that was significantly prevented by pharmacological CaM inhibition (On-line Fig. S4A, B).

Further studies in NRVM overexpressing GRK5 and Gq-CAM showed that CDZ treatment decreased basal levels of nuclear GRK5 as well as Gq-mediated accumulation (Fig. 3C, D). Additionally, in cells infected with Ad-GRK5 and treated with PE following 30 min of CDZ pretreatment, significant decreases in nuclear GRK5 were found–both basally and after α_1_AR stimulation (On-line Fig. S4C, D).

Using immunofluorescence, we further visualized the subcellular localization and nuclear translocation of endogenous GRK5. NRVM were infected with Ad-LacZ or Ad-Gq-CAM. On the second day following infection, cells were treated with DMSO or CDZ for 1 hr, fixed, and stained for GRK5 (Fig. 3E). Untreated cells expressing Lac-Z showed a diffuse distribution of GRK5 with some enrichment within the nuclei, while myocytes expressing Gq-CAM displayed a robust translocation of GRK5 to nuclei (Fig. 3E). CDZ treatment blocked movement of GRK5 into the nucleus, with myocytes retaining their diffuse staining pattern (Fig 3E, bottom row).

We further explored CaM-driven nuclear translocation of GRK5 after Gq-coupled receptor activation in myocytes by using W7, an alternative pharmacological inhibitor of CaM. W7 also strongly antagonizes activated CaM, but deviates in downstream effects compared to CDZ [39]. NRVM were treated with W7 in an analogous experiment to Fig. 3C. Nuclei isolated from cells after 1 hr of W7 treatment showed significantly decreased GRK5 accumulation basally (DMSO: 3.64±0.74; W7∶1.68±0.16, p<0.01) and following Gq-CAM stimulation (DMSO: 7.53±0.52; W7∶1.60±0.09, p<0.001) (On-line Fig. S4E, F).

Since data in Fig. 1 suggest that membrane GRK5 may act as the pool of this kinase shuttling to the nucleus after select Gq-coupled receptor activation, we paired TIRF microscopy and CDZ-treated AdRbM. Inhibition of CaM by CDZ restricts nuclear accrual of GRK5. Due to the likelihood of translocation by GRK5 from the plasma membrane to the nucleus, we were curious about the effects of CDZ on GRK5 at the membrane level. Similar to the TIRF experiments in Fig. 1D, AdRbM were infected with GRK5-GFP. Thirty minutes prior to imaging, cells were treated with CDZ and incubated at 37°C. Cardiomyocytes were imaged by TIRF microscopy using the same protocol as Fig. 1D, with addition of PE (Fig 3F) or AngII (Fig 3G) at 120 sec. In the case of either agonist, CDZ pretreatment led to constant measured fluorescence, blocking the swift and sustained movement of GRK5 away from the plasma membrane seen under control (DMSO) conditions. Additionally, pretreatment with CDZ led to a 7% fluorescence increase in non-stimulated cardiomyocytes. This suggests that, basally, CaM affects the subcellular localization of GRK5, and, after PE- or AngII-stimulation, CaM mediates the movement of this kinase off the plasma membrane.

### CaM-Binding to a Site on the Amino-Terminal Domain of GRK5 Directs its Nuclear Translocation

**Figure 4:**
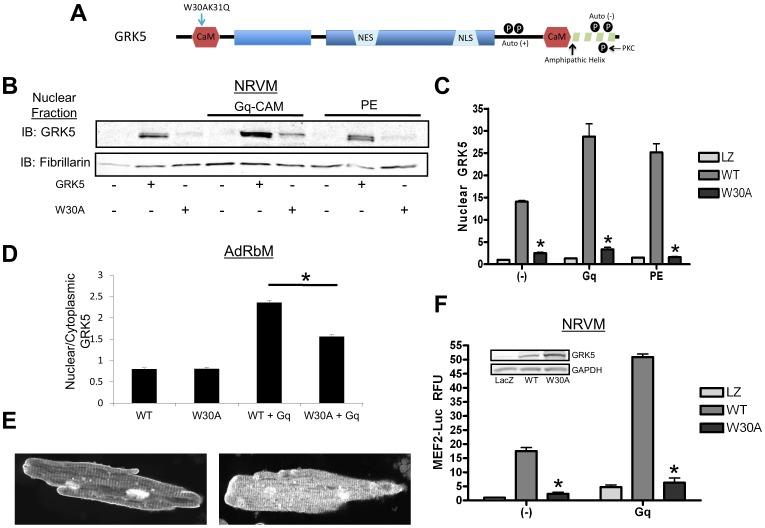
A mutant GRK5 (W30AK31Q) unable to bind CaM at its N-terminal CaM binding site displays less nuclear accumulation following Gq or PE stimulation. (**A**) Cartoon of GRK5’s structure illustrating pertinent domains and regulatory sites. (**B**) NRVM infected with Ad-GRK5 or Ad-GRK5W30A were stimulated with Ad-Gq-CAM (48 hr) or PE (1hr). Cells were then harvested by subcellular fractionation and immunoblotted for GRK5. (C) Quantitative analysis of (**B**) normalized to fibrillarin and reported as fold change over baseline. *p<0.001 v. WT GRK5, one-way ANOVA with a Bonferroni correction, n = 4. (**D**) AdRbM were co-infected with an adenovirus expressing either WT GRK5 tagged with GFP or GRK5 W30A tagged with GFP and Ad-Gq-CAM. Following an overnight culture, cells are imaged by confocal microscopy. Fluorescence within the nucleus was measured and normalized to cytoplasmic fluorescence. *p<0.001 v. WT GRK5+ Gq, one-way ANOVA with a Bonferroni correction, n = 4 (**E**) Images of representative myocytes showing WT GRK5-GFP (left) and GRK5W30A-GFP (right). (**F**) MEF2 activity in NRVM was measured using a luciferase assay system. Cells were co-infected with an adenovirus expressing a MEF2-luciferase reporter construct, Ad-LacZ, Ad-GRK5 or Ad-GRK5W30A and stimulated for 48 hr with the Ad-Gq-CAM virus. *p<0.001 v. WT GRK5, one-way ANOVA with a Bonferroni correction, n = 4, done in triplicate. Inset shows whole cell lysate of NRVM used in this experiment.

The above data is especially interesting because CaM is a known tight binding partner of GRK5. CaM binding inhibits GRK5 from acting on GPCRs, while retaining kinase activity towards soluble substrates [24]–[26], [40]. As shown in Fig. 4A, GRK5 has two CaM binding sites, one in each terminal domain. Prior analysis of these CaM-binding domains concluded that the N-terminal binding site appears most critical for CaM-mediated inhibition of GRK5 [25]. Two point mutations at amino acid residues 30 and 31 (W30A, K31Q) within the N-terminal CaM binding domain disrupt binding between GRK5 and CaM [25]. We created an adenovirus expressing GRK5 with these two point mutations (termed here as Ad-GRK5W30A) in order to examine the effects on CaM-mediated cellular localization of GRK5 after Gq-activating hypertrophic stimuli. First, NRVM were infected with Ad-LacZ, Ad-GRK5, or our new adenovirus, Ad-GRK5W30A. Some myocytes were also co-infected with Ad-Gq-CAM or treated with PE at 48 hrs post-infection. Myocytes co-overexpressing wild-type (WT) GRK5 and Gq-CAM or stimulated with PE showed a significant increase in nuclear GRK5 levels (Fig. 4B, C). In contrast, cells overexpressing GRK5W30A showed significantly less nuclear GRK5 at basal levels (2.5±0.34 vs. 14.1±0.47 for WT, P<0.05) and absolutely no change in response to Gq-CAM expression or PE treatment (Fig. 4B, C).

Differences in subcellular localization between WT GRK5 and GRK5W30A were also demonstrated in AdRbM. Cells were co-infected with Ad-Gq-CAM and Ad-GRK5-GFP or Ad-GRK5W30A-GFP and imaged by confocal microscopy. Nuclear fluorescence was normalized to cytoplasmic fluorescence and plotted in Fig. 4D. Cells expressing WT GRK5 displayed a 2.95±0.07 fold increase in nuclear:cytoplasmic fluorescence versus untreated, while W30A displayed significantly smaller increase (1.95±0.06 fold). Representative images of WT GRK5 (left) and W30A (right) are shown in Fig. 4E.

To determine any physiological significance of this lower nuclear accumulation due to diminished CaM binding to the N-terminal GRK5 mutant, we measured the effect of GRK5W30A overexpression on basal and Gq-mediated hypertrophic gene transcription. Previously, we have shown that nuclear GRK5 promotes hypertrophy as a Class II HDAC kinase via activation (de-repression) of the hypertrophic transcription factor, MEF2 [12]. Accordingly, we used a MEF2-luciferase reporter construct that expresses a promoter with multiple MEF2 binding sites and co-infected NRVM with Ad-LacZ, Ad-GRK5, or Ad-GRK5W30A. Induced myocytes were also co-infected with Ad-Gq-CAM. Normalized to baseline, overexpression of GRK5 without a stimulus increased MEF2-luciferase activity significantly (17.5±2.15 fold), while overexpression of GRK5W30A increased MEF2-luciferase activity minimally by only 2.38±0.99 fold (Fig 4F). Gq-CAM expression robustly increased MEF2 activity in control cells as well as in cells with concurrent WT GRK5 overexpression (50.9±1.86 fold). In contrast, overexpression of GRK5W30A led to no significant increase in MEF2 activity (Fig. 4F). Thus, restricting CaM’s ability to bind GRK5 at its N-terminal binding site limits nuclear accumulation of GRK5, eliminating its ability to facilitate hypertrophic gene transcription.

### CaM Binding to the N-Terminus of GRK5 Influences Response to Hypertrophic Agonists at the Plasma Membrane

**Figure 5:**
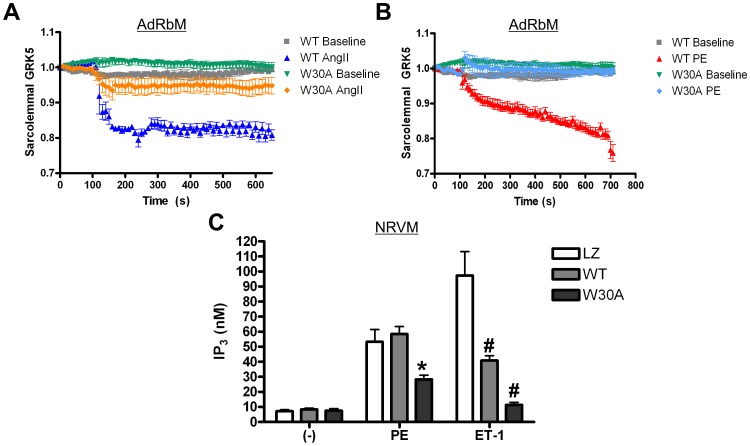
GRK5W30A displays increased plasma membrane association following agonist treatment and differential ability to desensitize GPCRs compared to WT. (**A**) AdRbM were infected with an adenovirus expressing GRK5-GFP or GRK5W30A-GFP and cultured overnight. Using TIRFM cells were imaged at 10 sec intervals for 700 sec. Baseline myocytes were untreated while stimulated myocytes were treated with either AngII (10 µM) (A) or PE (50µM) (**B**) at 120 sec. Fluorescence was normalized and reported to fold change versus baseline. n = 4 (**C**) Changes in GRK5 activity at the membrane was measured using an IP_1_ ELISA to determine changes in desensitization. NRVM were infected with Ad-LacZ, Ad-GRK5 or Ad-GRK5W30A. After 48 hours, cells were stimulated with PE or ET-1 for 2 hr, then assayed for IP_3_ generation via IP_1_ ELISA. *p<0.01 v. LacZ PE and WT PE, #p<0.01 v. LacZ ET-1, one-way ANOVA with a Bonferroni correction, n = 3, done in duplicate.

Our TIRF microscopy experiments above (Fig. 1D) suggest that specific hypertrophic Gq-coupled agonists induce GRK5 movement from the plasma membrane to the nucleus. Further, this recruitment can be disrupted by pharmacological CaM inhibition. The necessity of CaM binding to GRK5 at the plasma membrane was further reinforced by TIRF microscopy experiments using a GFP-tagged GRK5W30A mutant. AdRbM were infected with Ad-GRK5W30A-GFP and then stimulated with either AngII or PE. Without this N-terminal CaM-binding site, plasma membrane-associated GRK5W30A exhibited a limited, non-significant decrease in sarcolemmal fluorescence as a response to AngII (Fig. 5A). This diminished response to agonist was even more evident in PE-stimulated cardiomyocytes where there was no change in sarcolemmal fluorescence after PE application (Fig. 5B). Thus, CaM binding N-terminally is required for dissociation of GRK5 from the plasma membrane after hypertrophic stimulation.

The above deviations in subcellular localization between WT GRK5 and GRK5 W30A may lead to differences in GRK5’s canonical function - desensitization of GPCRs. In fact, it is interesting that PE and AngII, but not ET-1, caused wild-type GRK5 to leave the membrane and enter the nucleus. Thus, a question remains whether there are differences in GRK5’s kinase activity on these receptors in myocytes. We assessed this possibility by measuring the generation of downstream effectors of Gq, specifically IP_3_. NRVM were infected with Ad-LacZ, Ad-GRK5 or Ad-GRK5W30A and treated with either PE or ET-1, following which IP_3_ generation was quantified. In cells infected with Ad-LacZ, PE stimulation increased IP_3_ concentration from 7.15±1.66 nM to 53.3±14.1 nM while ET-1 treatment increased IP_3_ to 97.33±27.63 nM in the same cells (Fig. 5C). PE stimulation also increased IP_3_ to a similar concentration 58.4±8.72 nM in NRVM infected with WT GRK5 but generated significantly less IP_3_ (40.85±5.54 nM) when treated with ET-1 compared to LacZ-infected cells (Fig. 5C). Thus, ET-1 receptors appear to be desensitized and uncoupled with GRK5 overexpression while the PE response is unaffected. While this finding does not represent physiological desensitization due to the overexpression of WT GRK5, it does coincide with earlier reports that cardiac α_1_ARs are not apparent *in vivo* substrates for GRK5 [41]. The finding that WT GRK5 is able to desensitize ET-1 receptors but not α_1_ARs mirrors our TIRF data, where treatment with PE, but not ET-1 leads to dissociation of GRK5 from the plasma membrane. Thus, it appears that CaM binding can occur downstream of receptors that are not targets of GRK5’s desensitizing activity while activation of receptors that are substrates for GRK5 do not alter the membrane binding or nuclear accumulation properties of this kinase. This signaling consequence down-stream of selective Gq-coupled receptor activation has not been previously found and leads to a novel mechanistic hypothesis – that CaM significantly influences GRK5 activity within the nucleus and not at the level of the membrane-embedded GPCR. This notion is further reinforced by the W30A TIRF experiments since expression of GRK5W30A, which stays on the membrane, can now desensitize α_1_ARs and more profoundly attenuate ET-1R signaling (Fig 5C). In other words, when the CaM-GRK5 interaction is crippled GRK5 activity at the membrane is enhanced even at non-physiological substrates and no nuclear activity is seen.

To determine if an additional Ca^2+^ and CaM sources may lead to this increased interaction in the nucleus, we explored whether the IP_3_ receptor, which has been shown to be a nuclear store of Ca^2+^ [18], [42] could be involved. This appears to be the case as data in NRVM shows that activation of the myocyte IP_3_ receptor increases Gq-mediated GRK5 nuclear accumulation while its inhibition leads to a loss of Gq’s effects on GRK5 nuclear levels (On-line Fig. S5).

### CaM Binding to the N-Terminus of GRK5 is an In Vivo Requirement for Nuclear Effects of GRK5 on Hypertrophy

**Figure 6:**
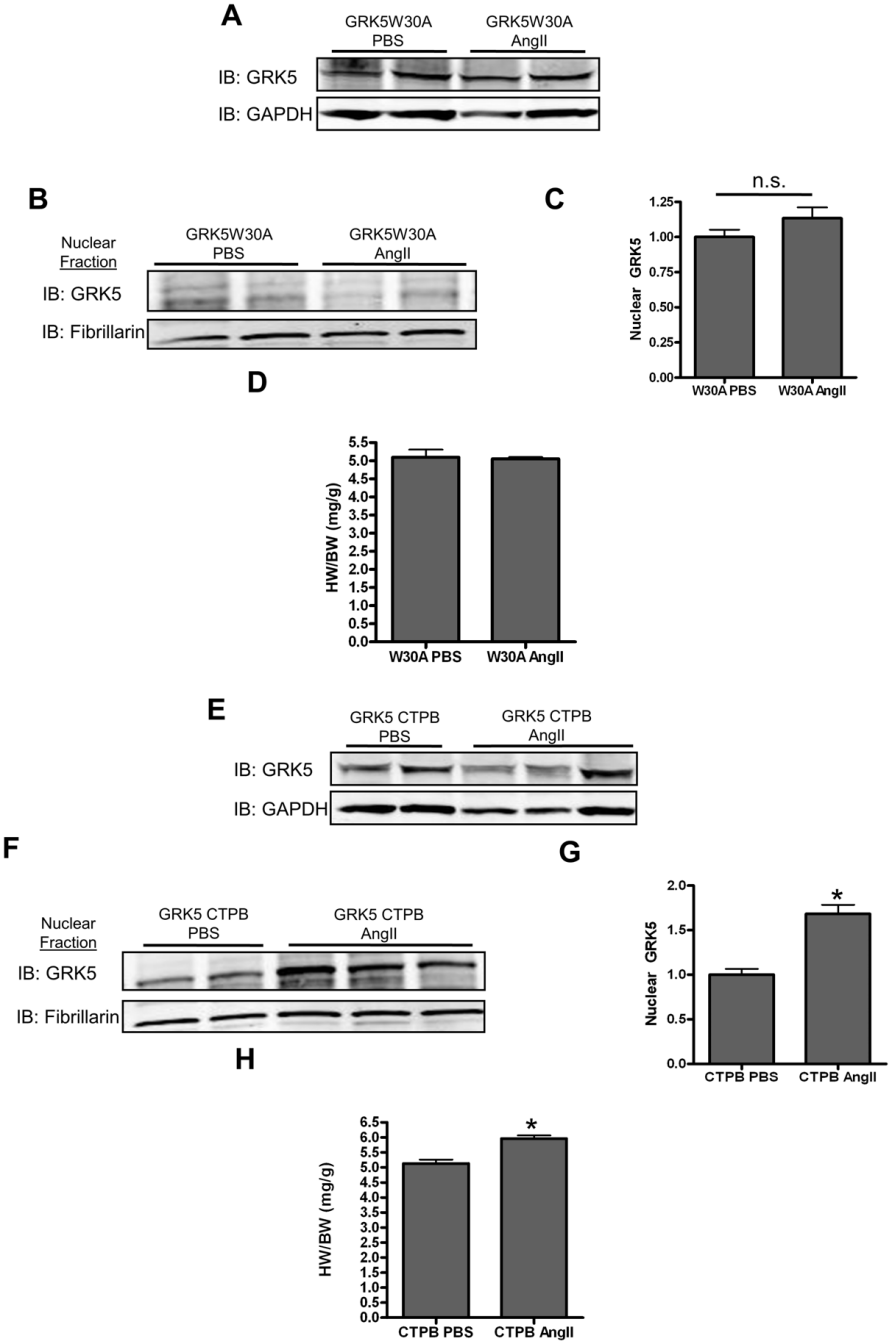
GRK5W30A demonstrates altered nuclear translocation *in vivo*. (**A**) Total cell lysates from GRK5KO injected with Ad-GRK5W30A into their LV free wall taken 10 days post-injection. (**B**) Nuclear lysates from mice with cardiac expression of only GRK5W30A that had received 72 hr of chronic PBS or AngII infusion were immunoblotted for GRK5. (**C**) Quantitative analysis of the nuclear lysates for nuclear GRK5 accumulation normalized to fibrillarin and reported as fold change. n = 8. (**D**) HW/BW ratio following 3 days of continuous PBS or AngII infusion for mice expressing GRK5W30A. (**E**) Total cell lysates from GRK5KO mice injected with Ad-GRK5 CTPB into their LV free wall taken 10 days post-injection. (**F**) Nuclear lysates from mice cardiac specific expression of only GRK5 CTPB that had received 72 hr of chronic PBS or AngII infusion were immunoblotted for GRK5. (**G**) Quantitative analysis of the nuclear lysates for nuclear GRK5 accumulation normalized to fibrillarin and reported as fold change. *p<0.05, student’s t test, n = 6 (H) HW/BW ratio following 3 days of continuous PBS or AngII infusion for mice expressing GRK5CTPB. *p<0.05, student’s t test, n = 6.

To further define the requirement and physiological significance of CaM in the nuclear localization and activity of GRK5, we tested whether GRK5-W30AK31Q could accelerate cardiac hypertrophy *in vivo*. Ad-GRK5W30A was directly injected into the LV free wall of global GRK5 knock-out (KO) mice, leading to robust expression of this mutant kinase alone after 7-10 days (Fig. 6A, and On-line Fig. S6). These mice were then treated to chronic infusion of AngII (200nM/kg/day) or PBS for 3 days, beginning 7 days following gene transfer. Mice were analyzed by echocardiography before and after treatment to measure cardiac function and dimensions. After 3 days, the animals were euthanized and hearts removed for analysis of hypertrophy and nuclear GRK5 levels. Importantly, and disparate from data in Tg-GRK5 mice in Fig. 2D, AngII treatment did not induce GRK5-W30A translocation to the nucleus of myocytes *in vivo*; levels were identical between PBS-treated and AngII-treated GRK5W30A-expressing KO mice (Fig. 6B, C). Further, these cardiac mutant mice did not have increased cardiac mass after 3 days of AngII, which we found in WT Tg-GRK5 (Fig. 2E). In fact, GRK5W30A-expressing mice had similar HW/BW ratios to mutant mice treated with saline (Fig. 6D).

As a crucial, further control for the above data, we used GRK5 KO mice and expressed another mutant GRK5 that cannot bind CaM at its C-terminal site but retains its N-terminal CaM binding site. This mutant, GRK5 CTPB, translocates to the nucleus of myocytes comparable to WT GRK5. In this experiment, GRK5 KO mice were injected with an adenovirus containing this mutant GRK5 (Fig. 6E) and then treated with AngII as above. Consistent with results in Fig. 2 for Tg-GRK5 mice, these mice, now expressing only GRK5 CTPB in their hearts, have significant accumulation of this kinase after AngII exposure as well as significantly increased HW/BW ratios (Fig. 6F–H). Together, these data indicate that CaM binding to the N-terminal site (W30A,K31Q) of GRK5 *in vivo* after a hypertrophic stimulus is an absolute requirement for the pathophysiological effects of this kinase, which occur after nuclear translocation.

## Discussion

Since its discovery, GRK5 has mainly been referenced in the context of its role in GPCR desensitization at the plasma membrane. An agonist-bound GPCR is rapidly phosphorylated by a GRK, triggering a conformational change and creating a docking site for β-arrestins. Internalization, followed by GPCR recycling or degradation, completes the desensitization process [4], [23]. Abundantly expressed in muscle, including the heart, GRK5’s predominant functions appear to encompass regulating cardiac inotropy and chronotropy downstream of the actions of catecholamines that bind and activate βARs. Up-regulated in failing myocardium, adverse effects of GRK5 initially have been attributed to βAR uncoupling and decreased inotropic reserve in HF [38], although GRK5 phosphorylation of some βARs can cause cardioprotection through transactivation of the epidermal growth factor receptors [28]. Recently, we addressed the role of endogenous GRK5 in the setting of cardiac hypertrophy. Ablation of this kinase conferred cardioprotection following the stress of pressure overload, blunting myocardial hypertrophy and delaying the onset of HF. Importantly, our results demonstrated an absolute requirement for cardiomyocyte GRK5 in the adaptive and maladaptive hypertrophic response [43].

Indeed, classically, GRK5’s primary association has been the sarcolemmal membrane, a fact thought to improve its GPCR targeting [44], [45]. However, increasing evidence has been amassed describing an extensive GRK5 “interactome.” New diverse substrates for GRK5 beyond GPCRs include: IκB [46], α-synuclein [47], p53 [48], [49], NFκB [50] and Hip [51]. Moreover, it has been demonstrated that GRK5 will accumulate in cellular locations distinct from the plasma membrane such as Lewy bodies [47] and centrosomes [49]. Most important to cardiac regulation has been the detection of GRK5 within the nucleus of cardiomyocytes and its novel role as a HDAC kinase [12], [14], [15].

**Figure 7:**
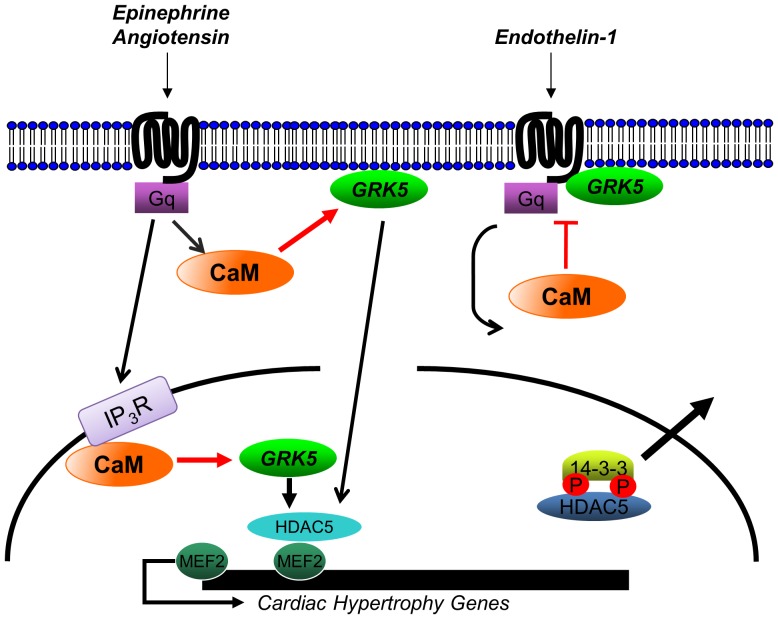
Cartoon depicting the select Gq-coupled receptor CaM-mediated translocation of GRK5 into the nucleus of cardiomyocytes. Gq activation due to catecholamines or AngII binding at the α_1_AR or AT-1R, respectively, causes CaM to bind GRK5 at its N-terminus, dislodging GRK5 from the plasma membrane. Via its NLS, GRK5 is directed to the nucleus where its interaction with CaM is stabilized by IP_3_R-regulated Ca^2+^ release. Once in the nucleus, GRK5 can act as an HDAC5 kinase, relieving repression of MEF2 and inducing hypertrophic gene transcription. In contrast, endothelin-1 binding leads to a selective interaction between the ET-1R substrate and the desensitizing GRK5. CaM cannot bind the kinase in this state, thus keeping GRK5 at the plasma membrane.

Nuclear GRK5 accumulation was first recognized as a potential downstream effect of HF generation in SHHF rats [14], [15]. We then identified GRK5’s role as an HDAC kinase, perpetuating negative effects on the stressed heart [12]. Nuclear localization and activity is unique to GRK5 among the GRK family. It appears to be an area ripe for potential therapeutic targeting that would prevent facilitation of maladaptive nuclear events while maintaining GPCR desensitizing capabilities. As such, we previously found that preventing GRK5 from entering the nucleus through mutation of its NLS ameliorated the accelerated hypertrophy and HF seen with increased cardiac GRK5 levels after ventricular pressure-overload [12]. Conversely, deletion of the kinase increases nuclear HDAC5, hindering cardiomyocyte hypertrophy [43]. Fully delineating the path of nuclear translocation would introduce the optimal place to disrupt this targeting, potentially leading to novel means of preventing HF development. Indeed, our current results, presented above, have led to the discovery of such a molecular target as we have proven the absolute mechanistic requirement for CaM in directing the nuclear translocation of GRK5 after select hypertrophic signaling. Our proposed mechanism is displayed in Fig. 7, with CaM acting as the primary upstream effector in promoting nuclear GRK5 accumulation after select hypertrophic Gq-coupled receptor activation. Based on our molecular signaling, imaging, and *in vivo* data, the interaction between GRK5 and CaM begins rapidly after receptor activation at the level of the membrane. Importantly, disrupting this interaction can block nuclear activity of GRK5, preventing maladaptive hypertrophy and HF.

The relationship between CaM and GRK5 has been previously described, although earlier *in vitro* studies presented no potential physiologic roles for this interaction [25], [26], [52]. GRK5 contains two CaM binding domains, one in each terminal region flanking the central catalytic domain (Fig. 4A). Data have shown that CaM binding prevents GRK5 from associating with plasma membrane and strongly inhibits its phosphorylation of GPCRs with an IC_50_ of 50nM [25], [26], [52]. Interestingly, while CaM decreases GRK5’s ability to phosphorylate membrane-bound substrates, such as GPCRs, it increases GRK5’s activity on cytosolic substrates [25]. One theory is that CaM binding lessens GRK5’s association with the membrane, increasing the distance between GRK5 and agonist-bound GPCRs. Thus, phosphorylation of these receptors is lessened or effectively inhibited. This observation is congruent with our data demonstrating CaM’s role in directing nuclear GRK5 translocation and activity after disrupting membrane association. GPCRs that do not drive nuclear GRK5, such as the ET-1R, may be preferred substrates for GRK5 compared to CaM, leading to substantial receptor desensitization and increased sarcolemmal retention. Conversely, αAR activation does drive rapid nuclear translocation, likely limiting GRK5’s GRK activity (as seen, Fig 5C). Consistent with this idea, the mutant GRK5 that cannot bind CaM at the N-terminal prevents GRK translocation from the membrane and enhances Gq-coupled receptor desensitization. Interestingly, the loss of N-terminal CaM binding also induces GRK5 to desensitize α_1_ARs, a receptor not targeted by wild-type GRK5 in the myocyte.

Importantly, away from the membrane, CaM-bound GRK5 appears rapidly in the nucleus of the Gq-activated myocyte where soluble nuclear molecules, such as HDAC5, become targets of its kinase activity (Fig. 7). This was evident as GRK5W30A does not accumulate in the nucleus after hypertrophic stimuli and loss of this HDAC kinase activity diminishes pathological gene transcription through MEF2. Moreover, mice with cardiac expression of only this CaM binding-deficient GRK5 mutant resulted in a resistance to AngII-mediated cardiac hypertrophy. Therefore, it is evident that eliminating the N-terminal CaM binding site in GRK5 abolishes the pathophysiological effects of increased nuclear GRK5 expression in the heart. Clearly, interruption of CaM binding to GRK5 may provide a new tool for preventing maladaption to hypertrophic stress and HF. Interestingly, our results with IP_3_ signaling show that the loss of CaM binding to GRK5 also increases the desensitization of hypertrophic Gq-coupled receptors. Theoretically, hypertrophic attenuation through the uncoupling of GPCR signaling could contribute synergistically, adding potential beneficial cardiac effects.

The GPCR effects of GRK5 show that ET-1 receptors are a selective substrate for GRK5 in cardiac myocytes and that CaM binding does not occur after activation of this Gq-coupled receptor. This is somewhat unexpected since CaM translocates to the nucleus in response to ET-1 [18]. One explanation is that the N-terminus of GRKs recognizes and binds to activated receptors [53], [54]. In this situation, the N-terminal of GRK5 is unavailable for CaM binding since activated ET-1 receptors are a preferred binding partner of GRK5 (Fig. 7). For other cardiac Gq-coupled receptors (α_1_AR and AT-1), GRK5 is not the primary desensitizing kinase and the sarcolemmal pool of GRK5 can be induced to translocate to the nucleus following receptor activation (Fig. 7). Therefore, the hypertrophic facilitation seen by increased myocyte GRK5 levels is selective depending on the stimulus, a mechanism analogous for other known HDAC kinases [30]. Of note, ET-1 has been shown to cause HDAC5 nuclear export through CaMKII, while PKD phosphorylates HDAC5 downstream of PE [30]. These results are consistent with GRK5-independent induction of hypertrophic gene transcription downstream of ET-1. *In vitro* studies that show increased nuclear export of HDAC5 by GRK5 only following AT_1_R activation [55] agree with our *in vivo* results. Of note, AngII was the most rapid inducer of GRK5 membrane movement, which may represent receptor-mediated pathophysiological effects of GRK5. Indeed, even at three days of AngII infusion, significant hypertrophy is evident by increased cardiac dimensions and greater HW/BW in mice with increased levels of GRK5. However, in our hands, we see that PE can also direct GRK5 nuclear translocation after dis-location from the sarcolemma causing early hypertrophy in Tg-GRK5 mice.

Of potential clinical importance, this segregated signaling downstream of Gq could be exploited when designing future pharmacological interventions. Selectivity for nuclear GRK5 activity may also explain discrepancies in the success of current HF treatments targeting Gq-coupled GPCRs. For example, AT_1_R antagonists (ARBs) such as Losartan, demonstrate efficacy in reversing cardiac hypertrophy in humans [56], [57]. Although the effects of ARBs are thought to be at least partly due to decreased blood pressure and cardiac load, patients treated with Losartan have attenuated hypertrophy accompanied by reduced cardiac fibrosis. This is interesting since genes responsible for both hypertrophy and fibrosis are regulated by MEF2 [58]. Concurrent with our data presented above, ARBs are likely to inhibit nuclear accumulation of GRK5 during cardiac stress and injury, allowing for repression of MEF2. In comparison, nuclear GRK5 is not a target for ET-1 and, interestingly, ET-1 receptor antagonists have shown less success in treating HF. Patients treated with ET-1_A_R and ET-1_B_R blockers showed no change in morbidity or mortality [59]. Additionally, no change in cardiac dimension was evident following a 24-week trial with an ET-1_A_R antagonist [60]. The differences between these trials and the ARB trials may lie in the distinct nuclear signaling events downstream of each Gq-coupled GPCR. Further studies can be done to explain whether the nuclear effects of GRK5 play a role in these critical translational and clinical findings.

It appears that, at the membrane, GRK5 demonstrates varying efficacy at specific GPCRs. This is an interesting finding with potential direct clinical implications since a recent human mutation has been uncovered and described for GRK5 [61]. This mutation, at amino acid residue 41 (a Q to L polymorphism), has been suggested to amplify GRK5-mediated desensitization of cardiac βARs. HF patients expressing this polymorphism do not respond well to β-blockers, but show less morbidity when β-blocker naïve, a finding explained by the possibility that this mutant GRK5 may act as an ”endogenous β-blocker” [61]. It is interesting to speculate that this alteration, proximal to the CaM binding site, could cause a change in the membrane dynamics of GRK5 after receptor activation that not only increases GRK activity at the membrane but lowers nuclear GRK5 activity, a possible contribution to the interesting positive findings in a HF population. This is something to test in further studies.

In summary, the current study defines the first physiological, and pathological, role of an interaction between CaM and GRK5 downstream of select Gq-coupled receptors. This dynamic interaction induces loss of GRK5 avidity for the plasma membrane and is an absolute requirement for the nuclear translocation of GRK5. Once in the nucleus, GRK5 imparts a crucial GPCR-independent activity to facilitate cardiac hypertrophy. When GRK5 is increased, as shown in human cardiac pathologies [8], [9], [11], it can induce maladaptative remodeling. Our findings indicate that disruption of CaM binding to the N-terminus of GRK5 may be a novel way to interrupt hypertrophic signaling and prevent HF through decreased nuclear HDAC kinase activity as well as improved GRK5 desensitizing capabilities on pathological GPCRs at the plasma membrane.

## Supporting Information

Figure S1 **Representative immunoblots of subcellular fractions in NRVM (A) or adult untreated c57/B6 mouse hearts (B).** Anti-β-tubulin was used as a marker for the non-nuclear compartment while anti-fibrillarin was used as a marker for the nuclear compartment. **(C)** Representative confocal images show sarcolemmal targeting of GRK5-GFP in AdRbM.
(TIF)

Figure S2 **AngII causes GRK5 accumulation in the nucleus of NRVM, while ET-1 and Iso do not.** (A) NRVM were infected with Ad-GRK5 (50 MOI). After 48 hr, cells were treated with 10 µM AngII for 5 different time points, harvested by subcellular fractionation. Nuclear fractions were immunoblotted for GRK5 and Fibrillarin. The amount of GRK5 in the nucleus was calculated by denistometry and normalized to Fibrillarin. Shown is a representative blot from 1 of 4 such experiments. **(B)** Nuclear Fractions in NRVM following a time course with Et-1 as described in **(A)** (100 nM). n = 3. **(B)** Nuclear Fractions in NRVM following a time course with Iso as described in **(A)** (10 µM). n = 4.
(TIF)

Figure S3 **Chronic infusion of Iso leads to no increase in nuclear GRK5.** Osmotic minipumps filled with PBS or Iso (60 mg/kg/day) were implanted into Tg-GRK5 mice. After 3 days, nuclei were isolated from the hearts of these mice and immunoblotted for GRK5 and fibrillarin. No change in the nuclear accumulation of GRK5 was seen.
(TIF)

Figure S4 **Inhibition of CaM blocks nuclear GRK5 accumulation after a physiological stimulus.** **(A)** NRVM were infected with Ad-GRK5. Two days after infection, cells were treated with DMSO or inhibitor: BIM1 (10 µM), Go6976 (10 µM), CDZ (10 µM) and KN93 (10 µM) for 30 min. Following inhibitor treatment, NRVM were stimulated with PE (50 µM) for 1 hr, then harvested and fractionated into nuclei. The isolated nuclei were analyzed by immunoblotting. **(B)** Immunoblots were quantitated by densitometry, normalized to fibrillarin, and reported as fold change over baseline. *p<0.01 v. untreated baseline; #p<0.001 v. CDZ, one-way ANOVA with a Bonferroni correction, n = 4. **(C)** NRVM were infected with Ad-LacZ or Ad-GRK5. 48 hr after infection, cells were pretreated with DMSO or CDZ for 30 min, then stimulated with PE for 1 hr. Cells were then harvested using subcellular fractionation and immunoblotted for GRK5. **(D)** Densitometric analysis for **(C)** with GRK5 normalized to fibrillarin and calculated as fold change over baseline. *p<0.05 v. DMSO GRK5; #p<0.01 v. DMSO GRK5+ Gq, one-way ANOVA with a Bonferroni correction, n = 4. **(E)** NRVM were infected with the same experimental design as Fig. 3C, but treated with W7 (10 µM) for 1 hr prior to harvest. **(F)** Densitometric analysis of **(E)** normalized to fibrillarin and reported as fold change over baseline. *p<0.01 v. DMSO treated GRK5, #p<0.001 v. DMSO GRK5+ Gq, one-way ANOVA with a Bonferroni correction, n = 4.
(TIF)

Figure S5 **Increasing IP_3_ in NRVM increases nuclear GRK5 accumulation.** (A) NRVM were infected with Ad-LacZ, Ad-GRK5 and Ad-Gq-CAM. 48 hr following infection, cells were stimulated with Adenophostin (10 µM), an IP_3_ receptor agonist, **(A)** or 2-APB (2 µM), an IP_3_ receptor antagonist, **(C)** for 1 hr, then harvested by subcellular fractionation. Nuclear fractions were immunoblotted for GRK5 and fibrillarin. **(B)** and **(D)** Densitometric analysis for nuclear GRK5 in **(A)** and **(C)**, respectively, normalized to fibrillarin and plotted as fold change over baseline. *p<0.001 v. untreated GRK5, #p<0.001 v. untreated GRK5+ Gq, one-way ANOVA with a Bonferroni correction, n = 4.
(TIF)

Figure S6 **Total GRK5 expression in GRK5KO hearts, either without infection, or 10 days following infection with Ad-GRK5W30A or Ad-GRK5CTPB.** Following adenoviral-mediated gene transfer, the hearts express equal amounts of the 2 GRK5 mutants.
(TIF)
