# Supplementary figures and images for: Nuclear Translocation of Cardiac G Protein-Coupled Receptor Kinase 5 Downstream of Select Gq-Activating Hypertrophic Ligands Is a Calmodulin-Dependent Process
Source: PLoS One. 2013 Mar 5;8(3):e57324. doi: 10.1371/journal.pone.0057324 (PMC3589474; doi:10.1371/journal.pone.0057324)

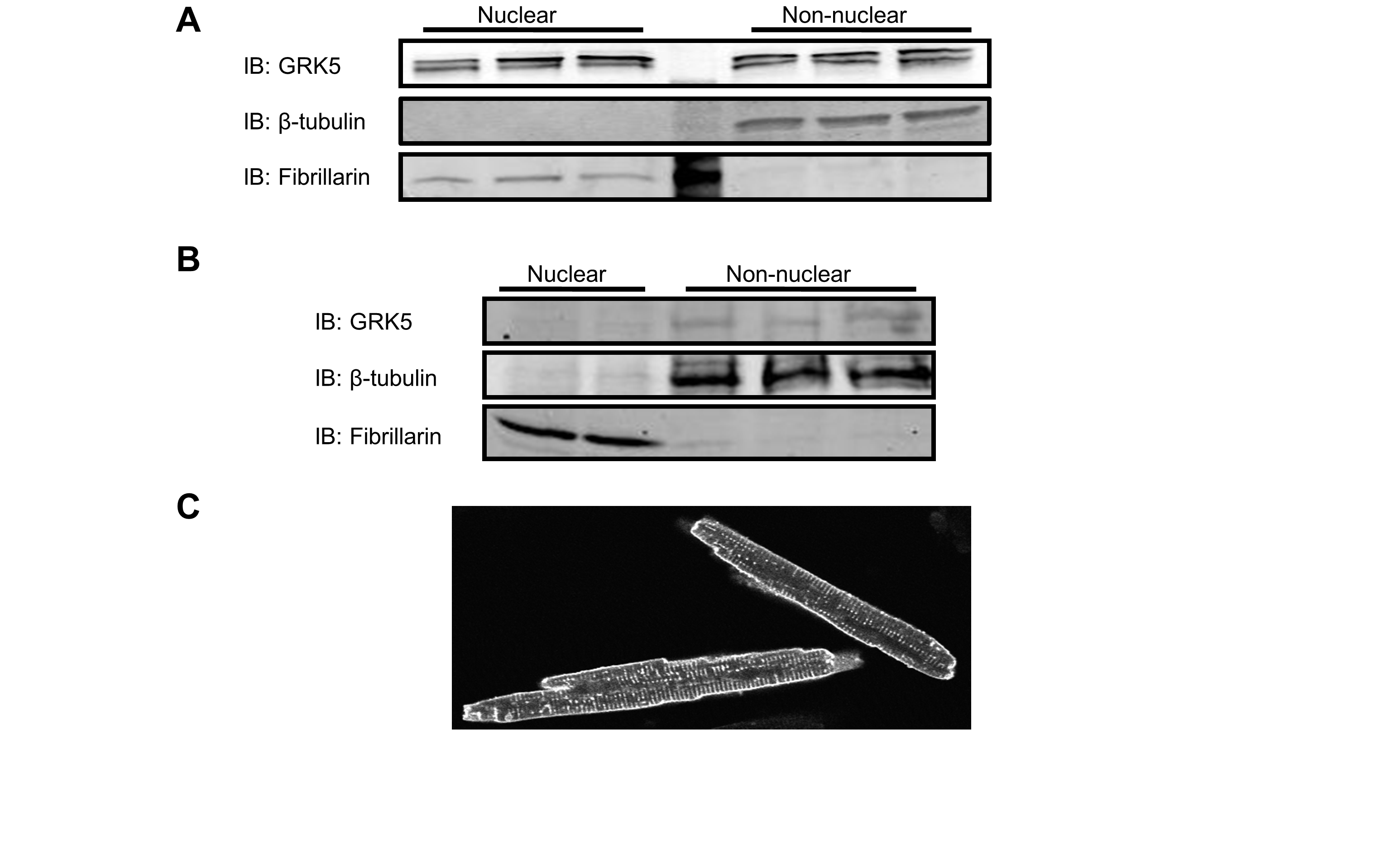

Supplement: Figure S1 — Representative immunoblots of subcellular fractions in NRVM (A) or adult untreated c57/B6 mouse hearts (B). Anti-β-tubulin was used as a marker for the non-nuclear compartment while anti-fibrillarin was used as a marker for the nuclear compartment. (C) Representative confocal images show sarcolemmal targeting of GRK5-GFP in AdRbM. (TIF) [file pone.0057324.s001.tif]

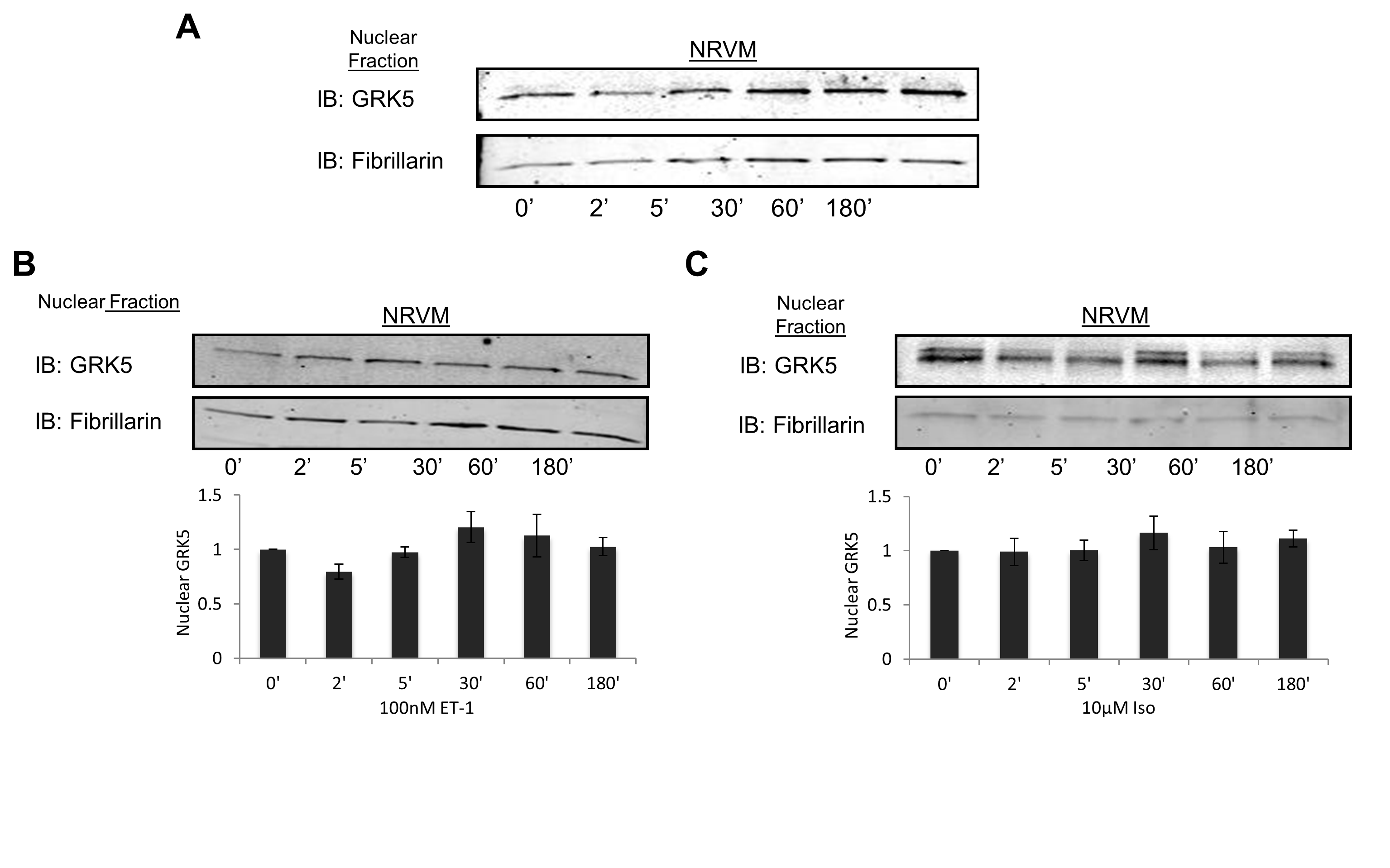

Supplement: Figure S2 — AngII causes GRK5 accumulation in the nucleus of NRVM, while ET-1 and Iso do not. (A) NRVM were infected with Ad-GRK5 (50 MOI). After 48 hr, cells were treated with 10 µM AngII for 5 different time points, harvested by subcellular fractionation. Nuclear fractions were immunoblotted for GRK5 and Fibrillarin. The amount of GRK5 in the nucleus was calculated by denistometry and normalized to Fibrillarin. Shown is a representative blot from 1 of 4 such experiments. (B) Nuclear Fractions in NRVM following a time course with Et-1 as described in (A) (100 nM). n = 3. (B) Nuclear Fractions in NRVM following a time course with Iso as described in (A) (10 µM). n = 4. (TIF) [file pone.0057324.s002.tif]

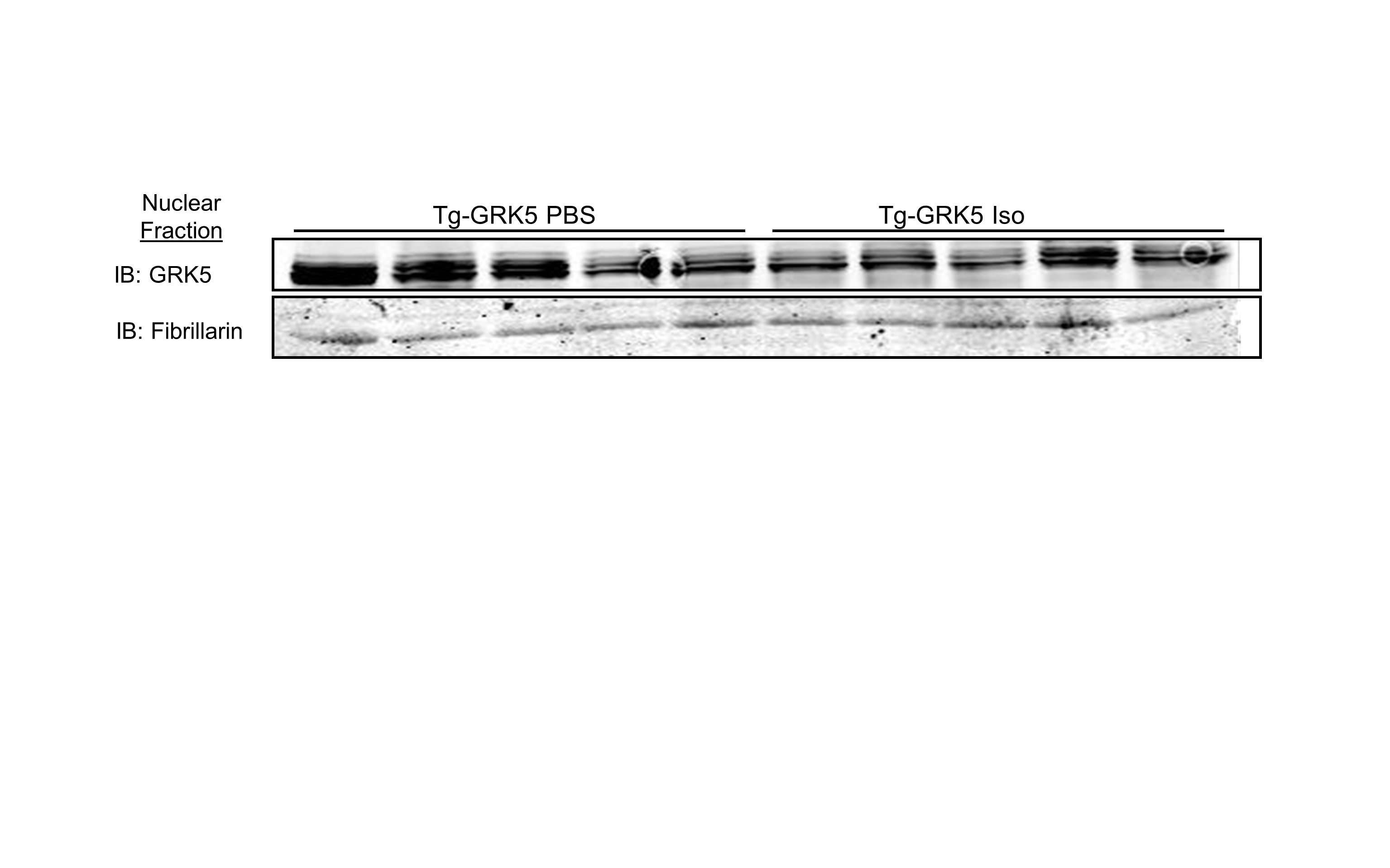

Supplement: Figure S3 — Chronic infusion of Iso leads to no increase in nuclear GRK5. Osmotic minipumps filled with PBS or Iso (60 mg/kg/day) were implanted into Tg-GRK5 mice. After 3 days, nuclei were isolated from the hearts of these mice and immunoblotted for GRK5 and fibrillarin. No change in the nuclear accumulation of GRK5 was seen. (TIF) [file pone.0057324.s003.tif]

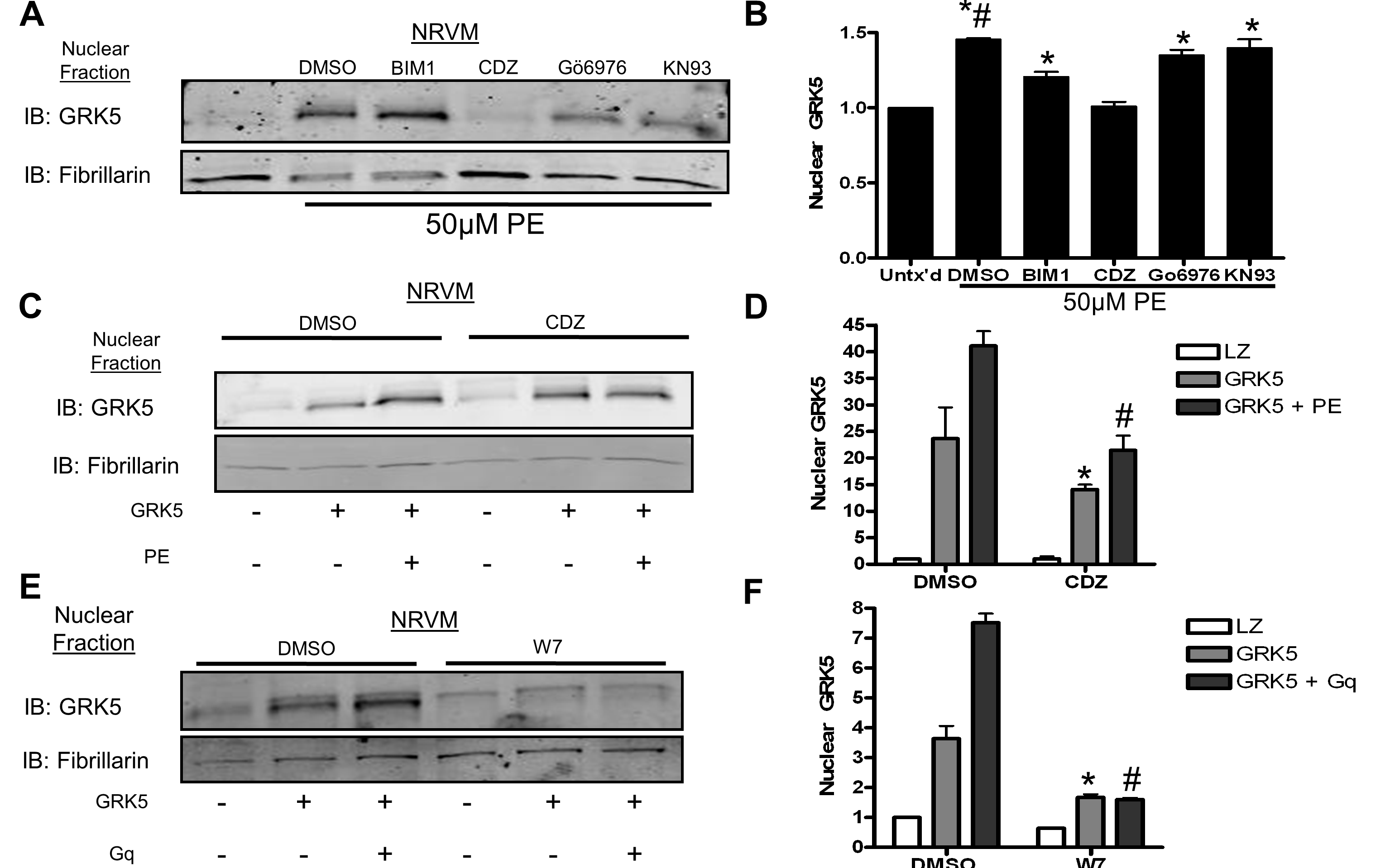

Supplement: Figure S4 — Inhibition of CaM blocks nuclear GRK5 accumulation after a physiological stimulus. (A) NRVM were infected with Ad-GRK5. Two days after infection, cells were treated with DMSO or inhibitor: BIM1 (10 µM), Go6976 (10 µM), CDZ (10 µM) and KN93 (10 µM) for 30 min. Following inhibitor treatment, NRVM were stimulated with PE (50 µM) for 1 hr, then harvested and fractionated into nuclei. The isolated nuclei were analyzed by immunoblotting. (B) Immunoblots were quantitated by densitometry, normalized to fibrillarin, and reported as fold change over baseline. *p<0.01 v. untreated baseline; #p<0.001 v. CDZ, one-way ANOVA with a Bonferroni correction, n = 4. (C) NRVM were infected with Ad-LacZ or Ad-GRK5. 48 hr after infection, cells were pretreated with DMSO or CDZ for 30 min, then stimulated with PE for 1 hr. Cells were then harvested using subcellular fractionation and immunoblotted for GRK5. (D) Densitometric analysis for (C) with GRK5 normalized to fibrillarin and calculated as fold change over baseline. *p<0.05 v. DMSO GRK5; #p<0.01 v. DMSO GRK5+ Gq, one-way ANOVA with a Bonferroni correction, n = 4. (E) NRVM were infected with the same experimental design as Fig. 3C, but treated with W7 (10 µM) for 1 hr prior to harvest. (F) Densitometric analysis of (E) normalized to fibrillarin and reported as fold change over baseline. *p<0.01 v. DMSO treated GRK5, #p<0.001 v. DMSO GRK5+ Gq, one-way ANOVA with a Bonferroni correction, n = 4. (TIF) [file pone.0057324.s004.tif]

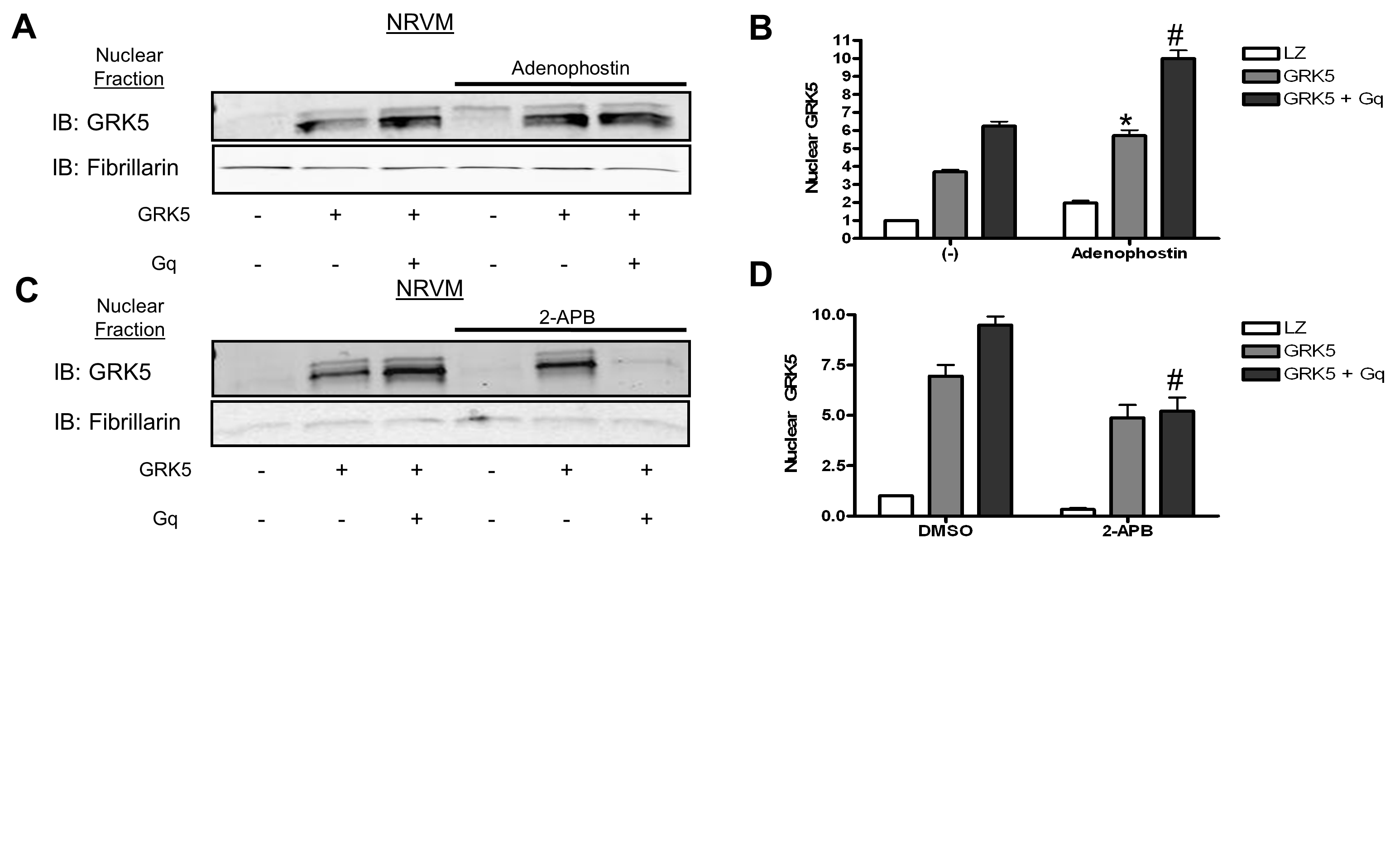

Supplement: Figure S5 — Increasing IP3 in NRVM increases nuclear GRK5 accumulation. (A) NRVM were infected with Ad-LacZ, Ad-GRK5 and Ad-Gq-CAM. 48 hr following infection, cells were stimulated with Adenophostin (10 µM), an IP3 receptor agonist, (A) or 2-APB (2 µM), an IP3 receptor antagonist, (C) for 1 hr, then harvested by subcellular fractionation. Nuclear fractions were immunoblotted for GRK5 and fibrillarin. (B) and (D) Densitometric analysis for nuclear GRK5 in (A) and (C), respectively, normalized to fibrillarin and plotted as fold change over baseline. *p<0.001 v. untreated GRK5, #p<0.001 v. untreated GRK5+ Gq, one-way ANOVA with a Bonferroni correction, n = 4. (TIF) [file pone.0057324.s005.tif]

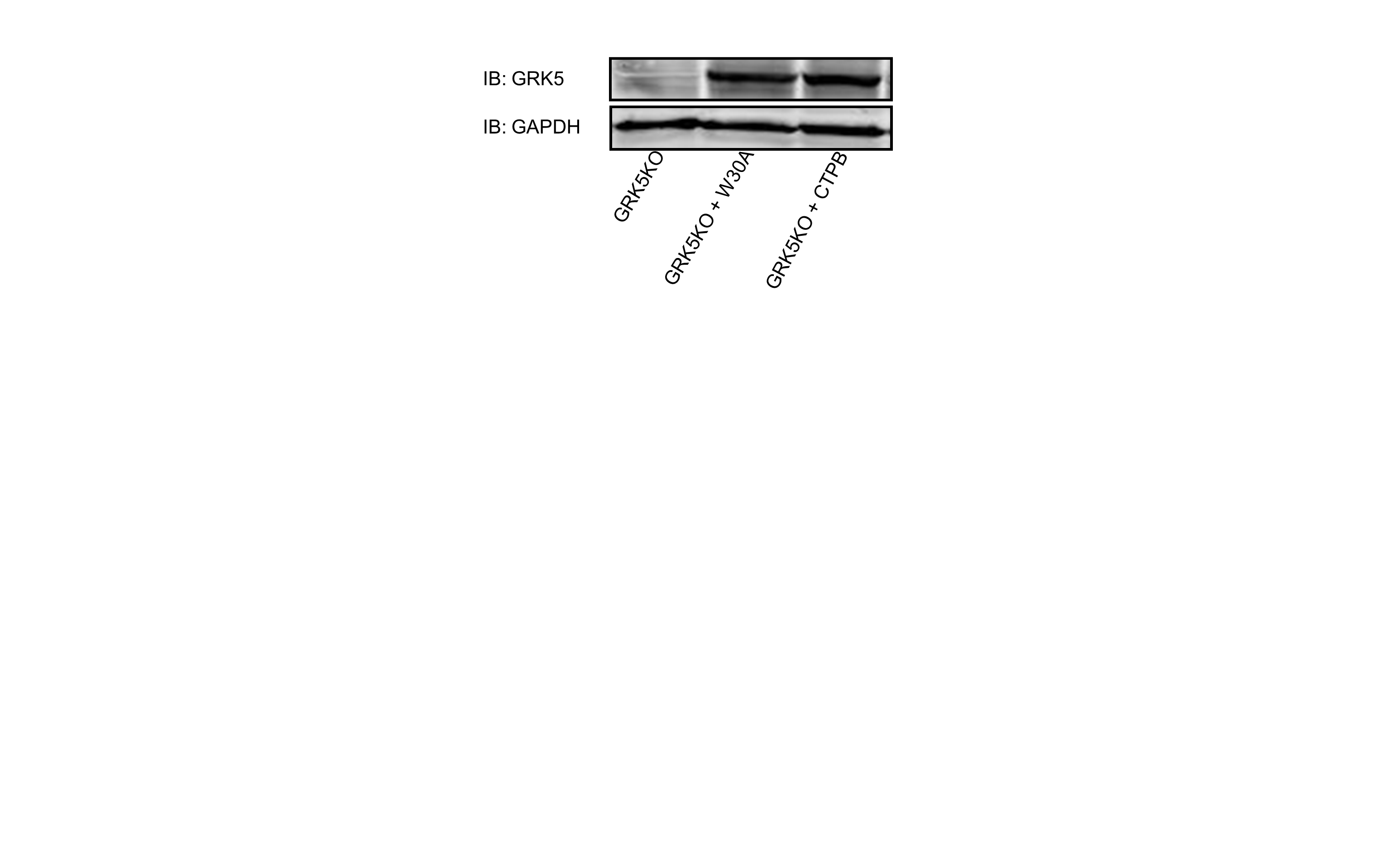

Supplement: Figure S6 — Total GRK5 expression in GRK5KO hearts, either without infection, or 10 days following infection with Ad-GRK5W30A or Ad-GRK5CTPB. Following adenoviral-mediated gene transfer, the hearts express equal amounts of the 2 GRK5 mutants. (TIF) [file pone.0057324.s006.tif]
